# Shengmai injection combined with conventional therapy in treating Adriamycin-related cardiotoxicity: A protocol for systematic review and meta-analysis of randomized controlled trials

**DOI:** 10.1097/MD.0000000000023084

**Published:** 2020-11-06

**Authors:** Lanchun Liu, Chao Liu, Lian Duan, Jing Bai, Qiyuan Mao, Wang Jie

**Affiliations:** a Guang’anmen Hospital, China Academy of Chinese Medical Sciences; b Beijing University of Chinese Medicine, Beijing, China.

**Keywords:** Adriamycin-related cardiotoxicity, drug-related side effects and adverse reactions, effectiveness, safety, Shengmai injection, study protocol, traditional Chinese medicine

## Abstract

**Background:**

Tumor is a common and frequently-occurring disease that seriously threatens human health, and is one of the main causes of death. Adriamycin (ADM) is the most commonly used and effective anti-tumor chemotherapeutics in clinical practice, but they can cause severe cardiotoxicity, which obviously limits their clinical application. Shengmai injection is a modern injection form of traditional Chinese medicine widely used for heart failure, myocardial infarction, cardiogenic shock, and cardiotoxicity patients in China. Therefore, we design this systematic review and meta-analysis to assess the effectiveness and safety of Shengmai injection for treating ADM-related cardiotoxicity.

**Methods:**

We will methodically search PubMed, EMBASE, Cochrane Library, Science Network, China National Knowledge Infrastructure, Wanfang Database, Chinese Journal Database, and China Biomedical Literature Database, in order to include randomized controlled trials which used Shengmai injection in treating ADM-related cardiotoxicity up to September 2020. The search strategies will use the following phrase: “Shengmai injection,” “Adriamycin,” “doxorubicin,” “cardiotoxicity,” “cardiomyopathy,” “randomized controlled trial.” The outcomes included cardiotoxicity rate, echocardiography, electrocardiogram, myocardial enzymes. Two researchers will independently select the study, extract the data and assess the quality by using Stata 14.0 and RevMan 5.3 software. The plan follows the preferred reporting items declared by the systematic review and meta-analysis plan, and the complete systematic review will follow the preferred reporting items for systematic reviews and meta-analysis (PRISMA) statement.

**Conclusion:**

The effectiveness and safety of Shengmai injection will be assessed in treating ADM-related cardiotoxicity which can give some evidence for clinical decision making.

**Trial registration number:**

INPLASY202090040

## 1 Introduction

Adriamycin (ADM), also known as doxorubicin (DOX), is one of the most commonly used anthracycline broad-spectrum antitumor drugs, and is the core drug of a variety of malignant tumor chemotherapy regimens.^[1,2]^ A wide range of clinical studies have shown that ADM presents serious toxicity, and its toxic effect on the heart is particularly obvious. In severe cases, it could cause heart failure, etc.^[3]^ The cardiotoxicity of ADM has been widely valued by medical workers since its discovery. Studies have shown that the ADM affinity with myocardial tissue is significantly higher than that with other tissues, which lead to the accumulation of ADM in myocardial cells. Meanwhile, myocardial tissue is more susceptible to ADM damage with a dose-dependent irreversible damage characteristic.^[4]^ This greatly limits the dosage of ADM used in clinical treatment, and increases the morbidity and mortality of cardiovascular disease in cancer treatment survivors. Once obvious cardiomyopathy appears, the prognosis of chemotherapy drugs is extremely poor.^[5]^ Because of the universality and severity of anthracycline heart injury, many patients with malignant tumors eventually die from serious cardiovascular complications, rather than the tumor itself. Therefore, the mechanism and prevention methods are the current common problem for both cardiovascular and tumor experts.

How to prevent and treat DOX-related cardiotoxicity is currently the focus of clinical research. Iron chelating agents have been proven to have cardioprotective effects. Among them, dextropropimine is not only proven to be a protective agent for anthracycline chemotherapy, but also to prevent myocardial damage.^[6]^ However, its expensive price and strict indications limit the clinical application of the drug. The study has also shown that dextroproimine has little effect on the overall progression-free survival of chemotherapy patients.^[7]^ Traditional antioxidants have a positive effect in protecting cardiomyocytes, reducing oxidative stress, and improving cardiomyocyte metabolism. However, no clinical trials have been proven that they can reduce the occurrence of anthracycline myocardial toxicity.^[8]^ SACCO confirmed that the angiotensin converting enzyme inhibitor Zonopril could reduce the myocardial damage caused by DOX through animal experiments, but the clinical effects were need to be further studied.^[9]^

Last several years, traditional Chinese medicine (TCM) has received more and more attention. Shengmai injection is a modern injection form of TCM, mainly composed of ginseng, Ophiopogon japonicus and Schisandra chinensis. Its mechanism of preventing the cardiotoxicity of anti-cancer drugs may be to enhance the contractility of myocardial cells, excite myocardium, and increase cardiac output.^[10]^ In recent years, many clinical reports have proved that Shengmai injection can improve the pumping function of the heart and the left ventricular ejection fraction (LVEF), reduce the cardiac afterload and the occurrence of arrhythmia.^[11]^ At the same time, a number of randomized controlled trials (RCT) about Shengmai injection in treating ADM-related cardiotoxicity have been carried out. Therefore, we hope to conduct a systematic review and meta-analysis to determine the effectiveness and safety about Shengmai injection in treating of ADM-related cardiotoxicity.

## 2 Review objectives

The main aims are to assess the effectiveness and safety of Shengmai injection vs conventional therapy in DOX-related cardiotoxicity with the improvement of cardiotoxicity rate, echocardiography, electrocardiogram, and myocardial enzymes. If possible, the incidence of side effects, allergies, and toxic reactions to analyze safety will be evaluated.

## 3 Methods

### 3.1 Protocol register

This protocol is based on the Systematic Review and Meta-Analysis Protocols recommendations,^[12]^ which can lead the process for protocols and assess the effectiveness of treatment. This study has been given a registration number in the International Platform of Registered Systematic Review and Meta-analysis Protocols (registration number: INPLASY202090040). When reporting the systematic review, the preferred reporting items for systematic reviews and meta-analyses protocols (PRISMA-P) will be carried out.^[13]^

### 3.2 Eligibility criteria

Participants, Intervention, Comparison, Outcomes and Study designs (PICOS) of inclusion and exclusion criteria are summarized as follows:

#### 3.2.1 Participants

(1) ***Included population.*** The standard of cardiotoxicity will be evaluated according to the acute and subacute toxicity standard of anti-cancer drugs established by WHO^[14]^; Adopt a first-line standard joint solution based on ADM; A RCT of Shengmai injection in preventing and treating of ADM-related cardiotoxicity.
(2) ***Exclusion criteria.*** Patients with severe arrhythmia, acute myocardial infarction, heart failure, severe neurosis, and menopausal syndrome in the past 6 months; Patients with severe primary diseases such as liver, kidney and hematopoietic system and mental illness.

#### 3.2.2 Interventions

The intervention group has received Shengmai injection treatment or combined with western medicine as a therapeutic intervention, and is limited to RCT for drug treatment. Shengmai injection is the only positive intervention in the treatment group compared with the control group, including placebo, no intervention, and anti-cancer drugs. There are no restrictions on the dosage and duration of medication but in studies comparing Shengmai injection with anti-cancer drugs, specifications, and dosage of anti-cancer drugs used in the treatment groups are the same as used in the control groups.

#### 3.2.3 Control

Control intervention measures can accept simple western medicine treatment, such as malignant lymphoma patients using CHOP regimen [CTX + ADM + VCR + Pred (Cyclophosphamide + Hydroxyldaunorubicin + Oncovin + Prednisone)], breast cancer patients using CAF regimen [CTX + ADM + 5-FU (Cyclophosphamide + Adriamycin + Fluorouracil)], ovarian cancer patients using CAP regimen [CTX + ADM + DDP (Cyclophosphamide + Adriamycin + Cisplatin)], multiple bone marrow tumor patients treated with VAD chemotherapy [VCR + ADM + DEX (Vincristine + Adriamycin + Dexamethasone)], or as a blank control without any treatment. Once the RCT includes any form of external treatment of TCM therapy, the patient will be excluded, including but not limited to moxibustion, acupuncture, and other tests.

#### 3.2.4 Outcomes

(1) ***Primary outcome indicator.*** The standard of cardiotoxicity rate will be evaluated according to the acute and subacute toxicity standard of anti-cancer drugs established by WHO^[14]^: Grade 0 means normal; Grade I means asymptomatic, but with abnormal heart signs; Grade II represents transient insufficiency of heart function, but does not require treatment; Grade III represents symptomatic, insufficient cardiac function, treatment is effective; Grade IV represents symptomatic, insufficient heart function, treatment is ineffective.
(2) ***Secondary outcome indicator.*** Echocardiography: mainly record LVEF which provided as a percentage (%); Electrocardiogram: arrhythmia after chemotherapy, ST- segment changes, T wave changes, QRS low voltage are recorded as ECG abnormalities; Myocardial enzymes: the grading standard of cardiac troponin I (cTnI) is based on the cut-off value recommended by the International United Chemistry and the World Standardization Committee^[15]^: the normal value of cTnI < 0.15ng/ml, 0.15 to 0.5 ng/ml indicates mild myocardial injury, and >0.5 ng/ml is severe myocardial damage.

#### 3.2.5 Study designs

The language included in the study is Chinese or English and will be an RCT. All the nontherapeutic research, animal experiments, case reports, review literature, literature with no control group, duplicate publications, incorrect data, unclear outcome effect, wrong statistical method, the measurement data without the mean and standard deviation will be excluded.

### 3.3 Data source

We will methodically search EMBASE, PubMed, Cochrane Library, Science Network, China National Knowledge Infrastructure, Wanfang Database, Chinese Journal Database, and China Biomedical Literature Database, for RCTs which used Shengmai injection in relieving ADM-related cardiotoxicity up to September 2020. including conference papers, dissertations, chapters in monographs and other gray literature. Missing data or other detailed information will be processed by contacting research investigators via email. In addition, relevant journals will be searched manually to track down the included articles. Medical Subject Heading or key words such as “shengmai injection” or “cardiotoxicity” or “doxorubicin” or “Adriamycin” will be used and the Chinese form of the above terms *(“sheng_mai,” “sheng_mai_zhu_she_ye,” “xin_zang_du,” “xin_ji_du,” "xin_ji_sun_shang,” “a_mei_su,” “duo_rou_bi_xin,” “en_huan)”* will be used for Chinese searches. Table 1 shows examples of specific searches for PubMed and China National Knowledge Infrastructure.

**Table 1:** Search strategies.

Database	Number	Search terms
Pubmed	#1	shengmai injection [Title/abstract] OR shengmai [Title/abstact] OR sheng mai [Title/abstract]
	#2	cardiotoxicity [Mesh] OR cardiotoxicity [Title/abstract] OR cardiac toxicity [Title/abstract] OR toxicity, cardiac [Title/abstract]
	#3	doxorubicin [Mesh] OR doxorubicin [Title/abstract]
	#4	Adriamycin [Title/abstract] OR farmiblastina [Title/abstract] OR ribodoxo [Title/abstract] OR rubex [Title/abstract] OR adriblastin [Title/abstract] OR adriblastine [Title/abstract] OR adriblastina [Title/abstract] OR adrimedac [Title/abstract] OR myocet [Title/abstract] OR onkodox [Title/abstract]
	#5	#3 OR #4
	#6	#2 AND #5
	#7	#1 AND #6
CNKI	#1	(subject) (*‘sheng_mai’* OR *‘sheng_mai_zhu_she_ye’*)
	#2	(subject)(*‘xin_zang_du’* OR *‘xin_ji_du’* OR *‘xin_ji_sun_shang’*)
	#3	(subject)(*‘a_mei_su’* OR *‘duo_rou_bi_xin’* OR *‘en_huan’*)
	#4	#2 AND #3
	#5	#1 AND #4

### 3.4 Studies and data extraction

#### 3.4.1 Studies selection

**Figure 1:**
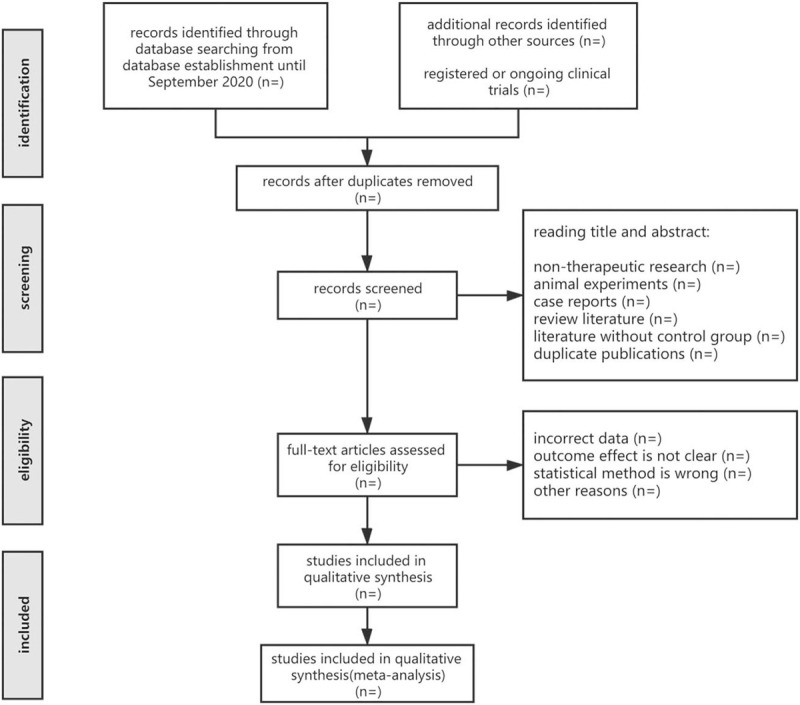
Preferred reporting items for systematic reviews and meta-analysis flow diagram.

Literature management will be carried out through Noteexpress software which will be firstly used to screen for repeated studies, and then researchers will remove irrelevant studies by filtering the title, abstract and other related information. Then, the literature will be further screened and any controversial documents will be discussed after obtaining the full text. Two professionally trained reviewers (Lanchun Liu and Chao Liu) will independently screen the documents in more detail, and then read and re-screen the full text of the articles that meet the inclusion criteria. In case of disagreement, the third reviewer (Lian Duan) participated in the discussion and resolved. The flow chart of literature screening is revealed in Figure 1.

#### 3.4.2 Data extraction

The review team will generate an extraction form after discussion. Two review authors (Jing Bai, Qiyuan Mao) will simultaneously conduct data extraction exercises on the basis of inclusion and exclusion criteria. Once any disputation is found, it will be solved by discussion with the third party (Lanchun Liu, Chao Liu). When necessary, the missing data or additional details will be handled by contacting the study investigators by E-mail. The content of data extracted from the original research is as follows:

(1) Fundamental features: research title, first author's name, year of publication, country or nation.
(2) Studies information: sample size, study design, explanation for withdrawal or dropout, follow-up duration.
(3) Participants’ information: sex distribution, age, severity of illness, comorbidity, diagnostic criteria, baseline level.
(4) Information of intervention group: intervention methods, duration, dose, and frequency.
(5) Information of control group: The original study needs to describe the detailed chemotherapy regimen, the packaging and color are the same as the therapy group to make sure neither the participant nor the researcher can distinguish.
(6) Outcome indicators: outcome measurement method, statistics of cardiotoxicity rate, echocardiography, electrocardiogram, and myocardial enzymes, safety indicators, adverse effects.
(7) Risk of bias: methods used to generate the randomization, assignment hiding, blinding of researchers and participants, allocation concealment, selective reporting, incomplete result data, other bias.
(8) Other research information: conflicts of interest, funding status.

### 3.5 Risk of bias assessment

Two reviewers (Lanchun Liu, Duan Lian) will separately identify the risk of bias according to Cochrane Collaboration Network Risk Assessment Tool, which include 7 aspects: selection bias (random sequence generation, allocation concealment), performance bias (blinding of participants and personnel), attrition bias (incomplete outcome data), detection bias (blinding of outcome assessment), reporting and other bias. Each research of bias situation will be judged by “low risk,” “unclear” and “high risk.”^[16]^ Differences between 2 review authors on the risk of bias will be adjudicated via investigation with the third party (Jing Bai, Qiyuan Mao). RevMan 5.3 software will be used to evaluate the risk of bias assessment chart. If important data on the study design is found to be missing, the corresponding author will be contacted via e-mail, phone or fax included in the published protocol or original study.

### 3.6 Data analysis and synthesis

Meta-analysis will be performed when the included trials have uniform and synthesizable clinical data.^[17]^ RevMan5.3 and STATA software will be used for meta-analysis. The dichotomous results will be expressed as a relative risk (RR) with a 95% confidence interval.^[18]^ If the measurement method is different, the continuous result will be expressed by the weighted standard mean difference (MD) which will be used while the measurement method is consistent. In this meta-analysis, echocardiography (EF, FS), myocardial enzyme (cTnI) will be presented as MD, while cardiotoxicity rate, electrocardiogram as RR. Cochran *Q* statistic and *I*^*2*^ statistic will be used to test heterogeneity.

For the whole analysis, if *P* ≥ .1 and *I*^2^ < 50%, then the literatures can be considered to be homogenous, which means the fixed effect model can be used for meta-analysis (Mantel-Haenzel method for RR and Inverse Variance for MD). If *P* < .1, *I*^2^ ≥ 50%, it can be considered that the heterogeneity between multi-studies has to be taken into consideration, and the random-effects model will be used (D-L method). *P* < .05 will be considered to be of statistically significance. If descriptive analysis of heterogeneity cannot be judged, no meta-analysis will be performed.

### 3.7 Subgroup analysis

In the case of sufficient data, if there is any heterogeneity between the studies, we will conduct a subgroup analysis. The subgroup analysis will be based on different cancer types, the year of publication, different races, control measures, comorbidities, measurement methods to see if there are influences in the outcome indicators.

### 3.8 Sensitivity analysis

The sensitivity analysis of meta-analysis is mainly to judge whether the decision of each step is sound and whether it will affect the result of the merger. The sensitivity analysis of the main outcome will use Stata 14 software, to detect the stability of meta-analysis. This can also be achieved by removing each study individually. If the heterogeneity is significantly reduced after deleting a study, the study is considered to be the main source of heterogeneity which should be further read and evaluated.

### 3.9 Publication bias

If the results of the meta-analysis include 10 or more articles, we will use a funnel chart to test the risk of publication bias. Quantitative methods will be used to help analyze publication bias by Egger test. Import the data into STATA software, enter the command “gen logor = log(_ES)” to define log (OR) which means the logarithm of the OR value. Enter the command “gen selogor = selog_ES” to define the standard error of the log (OR), turn it into a normal distribution and use the meta funnel command to draw a funnel chart. Use GRADEprofiler software to evaluate the quality of evidence, mainly for the grading of interventional evidence.

### 3.10 Ethics

Ethical approval is not required because the review is based on secondary studies of published literature and all data used in this study will be anonymous. The findings will be disseminated through a public issue journal, to inform both evidence-based medical evidence and decision-making clinical practice on Shengmai injection and cardiotoxicity.

## 4 Discussion

Anthracycline anti-tumor antibiotics are a kind of anti-tumor spectrum and powerful drugs, which are widely used in malignant solid tumors and hematological malignancies.^[19]^ The mechanisms of cardiotoxicity caused by such drugs include the effects of free radicals, calcium overload, mitochondrial damage, and cell apoptosis.^[20]^ The cardiotoxicity is the result of multiple factors. The side effects of chemotherapeutics belong to the category of drug toxicity. From the perspective of Chinese medicine, drug toxicity is a strong pathogenic factor.^[21]^ Drug toxicity is the most likely to hurt people's health. The situation is complicated and dangerous. The course of the disease is long-term and stubborn.

In recent years, clinical trials of TCM for intervention of ADM-related cardiotoxicity have increased greatly. In order to prevent and treat cardiotoxicity associated with DOX, more and more Chinese medicine programs are under development. Shengmai injection is a modern injection form of TCM, mainly composed of ginseng, Ophiopogon japonicus and Schisandra. Ginseng has obvious anti-hypoxia effects, and can resist myocardial ischemia and arrhythmia^[22,23]^; Ophiopogon japonicus water extraction can affect the electrophysiological characteristics of myocardium and has antiarrhythmic effects^[24]^; Schisandra contains a variety of active ingredients which can reduce the damage of myocardial cells, inhibit the infiltration of neutrophils, and protect myocardial ischemia-reperfusion injury.^[25]^ The application of Shengmai injection combined with chemotherapy has the effect of preventing and treating cardiotoxicity caused by chemotherapy. Its mechanism of preventing the cardiotoxicity of anti-cancer drugs may be to enhance the contractility of myocardial cells, excite myocardium, and increase cardiac output. In recent years, many clinical reports have proved that Shengmai injection can improve the pumping function of the heart and the LVEF of patients, reduce the cardiac afterload and the occurrence of arrhythmia.^[26,27]^

In conclusion, this study aims to assess the effectiveness of Shengmai injection on ADM-related cardiotoxicity. In order to determine the effectiveness of Shengmai injection, this study will conduct a comprehensive search of relevant literature that meets the standards, and provide a basis for clinical decision. Studies have shown that Shengmai injection can prevent and reduce cardiotoxicity caused by anthracyclines, especially acute cardiotoxicity. However, considering the complexity of TCM injection's composition and the patients’ individual differences, we should also draw attention to the safety of Shengmai injection. In this systematic review and meta-analysis, the effectiveness and safety of Shengmai injection in treating cardiotoxicity will also be analyzed.

### 4.1 Amendments

If there are any necessary modifications in this agreement, we will provide the date of each revision, characterize the specific changes, and give the reasons for each change.

## Author contributions

**Conceptualization:** Lanchun Liu, Chao Liu

**Data curation:** Lanchun Liu, Chao Liu, Lian Duan

**Formal analysis:** Jing Bai, Qiyuan Mao

**Methodology:** Lanchun Liu, Chao Liu, Lian Duan, Jing Bai, Qiyuan Mao

**Resources:** Lian Duan, Jing Bai

**Software:** Lanchun Liu, Chao Liu

**Funding acquisition:** Wang Jie

**Project administration:** Wang Jie

**Writing – original draft:** Lanchun Liu, Qiyuan Mao

**Writing – review & editing:** Chao Liu, Lian Duan

## References

[1] Moustaoui H Movia D Dupont N Tunable design of gold(III)-doxorubicin complex-PEGylated nanocarrier. The Golden doxorubicin for oncological applications. ACS Appl Mater Interfaces 2016;8:19946–57. 27424920 10.1021/acsami.6b07250

[2] Shanbhag S Ambinder RF Hodgkin lymphoma: a review and update on recent progress. CA Cancer J Clin 2018;68:116–32. 29194581 10.3322/caac.21438 PMC5842098

[3] Damiani RM Moura DJ Viau CM Pathways of cardiac toxicity: comparison between chemotherapeutic drugs doxorubicin and mitoxantrone. Arch Toxicol 2016;90:2063–76. 27342245 10.1007/s00204-016-1759-y

[4] Quiles JL Huertas JR Battino M Antioxidant nutrients and adriamycin toxicity. Toxicology 2002;180:79–95. 12324201 10.1016/s0300-483x(02)00383-9

[5] Abushouk AI Ismail A Salem AMA Cardioprotective mechanisms of phytochemicals against doxorubicin-induced cardiotoxicity. Biomed Pharmacother 2017;90:935–46. 28460429 10.1016/j.biopha.2017.04.033

[6] Cvetković RS Scott LJ Dexrazoxane: a review of its use for cardioprotection during anthracycline chemotherapy. Drugs 2005;65:1005–24. 15892593 10.2165/00003495-200565070-00008

[7] Swain SM Whaley FS Gerber MC Cardioprotection with dexrazoxane for doxorubicin-containing therapy in advanced breast cancer. J Clin Oncol 1997;15:1318–32. 9193323 10.1200/JCO.1997.15.4.1318

[8] van Dalen EC Caron HN Dickinson HO Cardioprotective interventions for cancer patients receiving anthracyclines. Cochrane Database Syst Rev 2011;2011:Cd003917. 10.1002/14651858.CD003917.pub3 18425895

[9] Sacco G Mario B Lopez G ACE inhibition and protection from doxorubicin-induced cardiotoxicity in the rat. Vasc Pharmacol 2009;50:166–70. 10.1016/j.vph.2009.01.001 19344651

[10] Zhang YK He WW Zhang H Observation on the efficacy of Shengmai injection in preventing acute cardiotoxicity of adriamycin in 40 cases. Chin Emerg Tradit Chin Med 2007;16:55–6.

[11] Mao JY Zhao ZQ Xu X Overview and prospects of research on the mechanism of Shengmai injection in the treatment of heart failure. Chin Emerg Tradit Chin Med 2007;16:216–7.

[12] Moher D Shamseer L Clarke M Preferred reporting items for systematic review and meta-analysis protocols (PRISMA-P) 2015 statement. Syst Rev 2015;4:1. 25554246 10.1186/2046-4053-4-1 PMC4320440

[13] Shamseer L Moher D Clarke M Preferred reporting items for systematic review and meta-analysis protocols (PRISMA-P) 2015: elaboration and explanation. BMJ (Clin Res Ed) 2015;350:g7647. 10.1136/bmj.g7647 25555855

[14] Du XJ Woodcock EA Little PJ Protection of neuronal uptake-1 inhibitors in ischemic and anoxic hearts by norepinephrine-dependent and -independent mechanisms. J Cardiovasc Pharmacol 1998;32:621–8. 9781931 10.1097/00005344-199810000-00015

[15] Barry E Alvarez JA Scully RE Anthracycline-induced cardiotoxicity: course, pathophysiology, prevention and management. Expert Opin Pharmacother 2007;8:1039–58. 17516870 10.1517/14656566.8.8.1039

[16] Higgins JP Altman DG Gøtzsche PC The Cochrane Collaboration's tool for assessing risk of bias in randomised trials. BMJ (Clin Res Ed) 2011;343:d5928. 10.1136/bmj.d5928 PMC3196245 22008217

[17] Liu M Design and Implementation Methods of Systematic Review Meta-Analysis. Beijing, China: People's Medical Publishing House; 2011.

[18] Li YP Practice of Evidence-Based Medicine. Beijing, China: People's Medical Publishing House; 2018.

[19] Octavia Y Tocchetti CG Gabrielson KL Doxorubicin-induced cardiomyopathy: from molecular mechanisms to therapeutic strategies. J Mol Cell Cardiol 2012;52:1213–25. 22465037 10.1016/j.yjmcc.2012.03.006

[20] De Beer EL Bottone AE Voest EE Doxorubicin and mechanical performance of cardiac trabeculae after acute and chronic treatment: a review. Eur J Pharmacol 2001;415:1–1. 11245845 10.1016/s0014-2999(01)00765-8

[21] Zheng HG Piao BK Discussion on the causes of side effects of radiotherapy and chemotherapy in traditional Chinese medicine. Chin J Basic Med Tradit Chin Med 2007;13:751–2.

[22] Yu C Study on the pharmacological effects of ginsenosides on cardiovascular system. Tianjin Pharmacy 2010;22:45–7.

[23] Wang TC Zhang H Research progress on the antiarrhythmic effects of ginseng saponin. Chin J Cardiac Pacing Electrophysiol 2004;18:309–10.

[24] Sun J Cai M Research progress on the chemical constituents and pharmacological effects of Ophiopogon japonicus. Chin Pharmacovigil 2010;7:681–3.

[25] Liu W Zhang Q Zhang CY Study on the effects of Schisandra on the cardiovascular system. J Beihua Univ 2011;12:47–9.

[26] Yang XL Clinical observation of Shengmai injection in preventing doxorubicin-related cardiotoxicity. China Med Guide 2008;6:203–5.

[27] Shen XM Zhu AQ Liu GH The effect of Shengmai injection on the cardiotoxicity of multi-course chemotherapy with epirubicin for breast cancer. Clin Med Res Pract 2020;5:128–30.

